# GNSS Precise Kinematic Positioning for Multiple Kinematic Stations Based on *A Priori* Distance Constraints

**DOI:** 10.3390/s16040470

**Published:** 2016-04-01

**Authors:** Kaifei He, Tianhe Xu, Christoph Förste, Svetozar Petrovic, Franz Barthelmes, Nan Jiang, Frank Flechtner

**Affiliations:** 1 School of Geosciences, China University of Petroleum (East China), Qingdao 266580, China; kfhe@upc.edu.cn; 2 State Key Laboratory of Geo-information Engineering, Xi’an Research Institute of Surveying and Mapping, Xi’an 710054, China; 3 Institute of Space Science, Shandong University, Weihai 264209, China; 4 German Research Centre for Geosciences (GFZ), Potsdam 14473, Germany; foer@gfz-potsdam.de (C.F.); sp@gfz-potsdam.de (S.P.); bar@gfz-potsdam.de (F.B.); flechtne@gfz-potsdam.de (F.F.); 5 Berlin University of Technology (TU Berlin), Berlin 10623, Germany; jiangnan1112@163.com

**Keywords:** airborne gravimetry, shipborne gravimetry, GNSS sensors system, precise kinematic positioning, *a priori* distance constraint, multiple kinematic stations

## Abstract

When applying the Global Navigation Satellite System (GNSS) for precise kinematic positioning in airborne and shipborne gravimetry, multiple GNSS receiving equipment is often fixed mounted on the kinematic platform carrying the gravimetry instrumentation. Thus, the distances among these GNSS antennas are known and invariant. This information can be used to improve the accuracy and reliability of the state estimates. For this purpose, the known distances between the antennas are applied as *a priori* constraints within the state parameters adjustment. These constraints are introduced in such a way that their accuracy is taken into account. To test this approach, GNSS data of a Baltic Sea shipborne gravimetric campaign have been used. The results of our study show that an application of distance constraints improves the accuracy of the GNSS kinematic positioning, for example, by about 4 mm for the radial component.

## 1. Introduction

Measuring the Earth’s gravity field is an important topic in many scientific and economic applications, such as geodesy, geophysics, explorations, geoid determination, and satellite orbit computation [1]. In this context, airborne and shipborne gravimetry play a very important role in recovering the Earth’s gravity field in the range of medium to high frequencies [2]. In that work area, precise kinematic positioning based on the Global Navigation Satellite System (GNSS) plays a significant role [3], since the state information of a kinematic platform (a ship or an airplane) carrying a gravimeter can be obtained independently from GNSS observations. Trajectory and attitude of such a kinematic platform are indispensable information for analyzing gravimetry data. The acceleration information derived from the GNSS position and/or velocity information for such a kinematic platform can be used to separate the disturbing kinematic accelerations from the gravitational signal. Therefore, the estimation of accurate state information for such a kinematic platform by precise GNSS positioning is a key factor for any successful implementation of airborne and shipborne gravimetry [4,5,6].

**Figure 1:**
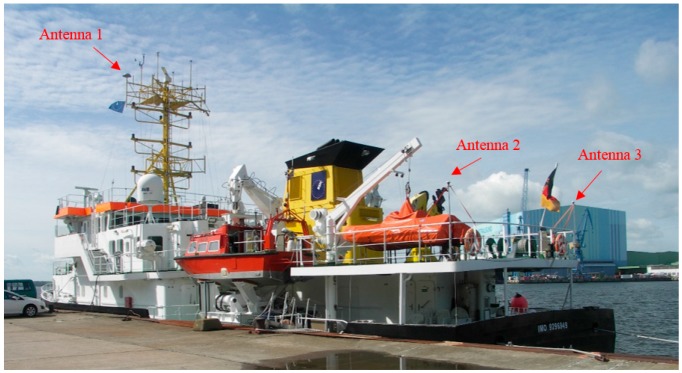
The ship used in the Baltic Sea gravimetric campaign and the positions of the three Global Navigation Satellite System (GNSS) receiving antennas.

In our study, we used the data of a shipborne gravimetric campaign on the Baltic Sea which was organized by the German Research Centre for Geosciences (GFZ) and the Federal Agency for Cartography and Geodesy (BKG). The gravimetric measurements were taken near Greifswald, Germany from 18 to 27 June 2013. The ship used for this campaign including the arrangement of the GNSS antennas installed thereon is shown in Figure 1. The positions of these antennas were surveyed with respect to the local reference frame of the vessel. Thus, the distances among the multiple GNSS kinematic antennas are known and can be used to improve the accuracy and reliability of the state estimates and to control their uncertainties. However, as explained later on, the distance used in this study was determined in a different way, *i.e.*, from GNSS observation.

For this purpose, a method of GNSS kinematic positioning based on multiple kinematic stations with multiple reference stations [7] was in a first step updated to use the known distances among the multiple GNSS kinematic antennas as *a priori* constraints within the corresponding state parameters adjustment. In a second step, the data of the mentioned Baltic Sea shipborne campaign have been processed with and without these constraints. Finally, we summarize the findings from our application of distance constraints for GNSS kinematic positioning.

## 2. Kinematic Positioning Based on *A Priori* Distance Constraints

The principle of the method of GNSS kinematic positioning with multiple kinematic and multiple reference stations [7] can be briefly described as follows: When double difference (DD) observation equations are formed, there is only one station that is used as a formal reference station. The other reference stations are processed formally as kinematic stations together with the actual moving kinematic stations, but, in contrast to the actual moving kinematic stations, *a priori* constraints based on the known station information are applied to other reference stations. In other words, their positions are known and treated as unchangeable. Without these constraints, they would actually be kinematic stations.

Based on the classic Kalman filter theory and the method of GNSS kinematic positioning with multiple kinematic and multiple reference stations, the principle of *a priori* distance constraints within the state parameters was developed as briefly described in the following.

### 2.1. Classic Kalman Filter

The system state equation and observation equation of GNSS kinematic positioning are generally expressed as [8]

(1) $$X_{i}=\Phi_{i.i-1}X_{i-1}+W_{i}$$

and

(2) $$L_{i}=A_{i}X_{i}+e_{i}$$

where $X_{i}$ and $X_{i-1}$ are m×1 state vectors at epochs $t_{i}$ and $t_{i-1}$, respectively. $\Phi_{i.i-1}$ is a m×m transition matrix from state $X_{i-1}$ to $X_{i}$, and $W_{i}$ is an m×1 error vector of system state model with zero mean and covariance matrix $\Sigma_{W_{i}}$. $L_{i}$ is a n×1 measurement vector at epoch $t_{i}$, $A_{i}$ is a n×m design matrix, and $e_{i}$ is a measurement error vector with zero mean and covariance matrix $\Sigma_{i}=\sigma_{0}^{2}P_{i}^{-1}$, where $\sigma_{0}^{2}$ is the theoretical variance of unit weight and $P_{i}$ denotes the weight matrix of observations.

The predicted state vector ${\bar{X}}_{i}$ and its covariance matrix $\Sigma_{{\bar{X}}_{i}}$ are denoted by

(3) $${\bar{X}}_{i}=\Phi_{i,i-1}{\hat{X}}_{i-1}$$

and

(4) $$\Sigma_{{\bar{X}}_{i}}=\Phi_{i,i-1}\Sigma_{{\hat{X}}_{i-1}}\Phi_{i,i-1}^{T}+\Sigma_{W_{i}}$$

The transition matrix $\Phi_{i.i-1}$ of the state vector $X_{i}$ and the covariance matrix $\Sigma_{W_{i}}$ of the error vector $W_{i}$ of the Kalman filter system state model are described in the literature [8,9,10].

The error equations for the predicted state vector and the measurement vector are

(5) $$V_{{\bar{X}}_{i}}={\hat{X}}_{i}-{\bar{X}}_{i}$$

and

(6) $$V_{i}=A_{i}{\hat{X}}_{i}-L_{i}$$

where $V_{{\bar{X}}_{i}}$ and $V_{i}$ denote the estimators of the vectors $W_{i}$ and $e_{i}$, respectively.

According to the classical Kalman filter theory [8,10,11,12,13,14], the state estimate at epoch *i* can be expressed as

(7) $${\hat{X}}_{i}={\bar{X}}_{i}+\Sigma_{{\bar{X}}_{i}}A_{i}^{Τ}{\left(A_{i}\Sigma_{{\bar{X}}_{i}}A_{i}^{Τ}+\Sigma_{i}\right)}^{-1}\left(L_{i}-A_{i}{\bar{X}}_{i}\right)$$

with its covariance matrix $\Sigma_{{\hat{X}}_{i}}$,

(8) $$\Sigma_{{\hat{X}}_{i}}=\left(I-\Sigma_{{\bar{X}}_{i}}A_{i}^{Τ}{\left(A_{i}\Sigma_{{\bar{X}}_{i}}A_{i}^{Τ}+\Sigma_{i}\right)}^{-1}A_{i}\right)\Sigma_{{\bar{X}}_{i}}$$

where I is the identity matrix.

### 2.2. The Distance between Two Kinematic GNSS Antennas

When certain constraints in the GNSS kinematic state parameters of a multiple GNSS sensors system are given, they should be taken into account in order to improve the positioning accuracy and reliability. A typical example for such a constraint option is the known distance between two GNSS antennas mounted on a kinematic platform. This distance is

(9) $$d_{i}^{k_{1},k_{2}}=\sqrt{{\left(x_{i}^{k_{1}}-x_{i}^{k_{2}}\right)}^{2}+{\left(y_{i}^{k_{1}}-y_{i}^{k_{2}}\right)}^{2}+{\left(z_{i}^{k_{1}}-z_{i}^{k_{2}}\right)}^{2}}$$

where *k*_1_ and *k*_2_ are the kinematic stations (antennas) and (*x_i_*, *y_i_*, *z_i_*) their respective position vectors at the epoch *i*. The precision of this distance was determined from the error estimates of the measurements performed in order to determine it.

There are several approaches to deal with *a priori* constraints in Kalman filter applications [15], including linearized constraints [16], nonlinear constraints [17], and timevarying constraints [18]. In this study, a specific kind of *a priori* distance constraint is applied for the GNSS kinematic positioning, as described in the following section.

### 2.3. The A Priori Distance Constraints

Due to the offset of the GNSS antenna phase center, the distances between GNSS antennas are difficult to measure precisely in a simple way (for instance by using a ruler). Therefore, in this study, DD processing on ultrashort baselines is applied to measure the distances among GNSS antennas with millimeter accuracy. These precisely known distances of kinematic antennas are used as the *a priori* constraints by means of the so-called pseudo observation method*,* *i.e.*, by expressing the constraints as highly weighted observations that strengthen the structure of the measurement space directly.

When multiple kinematic stations are mounted on a moving platform, the linearized constraint equation can be written as

(10) $$D=B_{i}X_{i}+\varepsilon$$

where D denotes the u×1 distance constraint vector at every epoch i, and $B_{i}$ is a u×m design matrix. $X_{i}$ is the m×1 unknown parameter vector at epoch i, and ε is a distance constraint uncertainty vector with zero mean and covariance matrix $\Sigma_{d}$.

To express the distance constraints among the kinematic antennas as observation equations, error Equation (10) can be combined with GNSS observation error Equation (6) of a classical Kalman filter as

(11) $$\left[\begin{array}{c} V_{i}\\ V_{i}^{d} \end{array}\right]=\left[\begin{array}{c} A_{i}\\ B_{i} \end{array}\right]{\hat{X}}_{i}-\left[\begin{array}{c} L_{i}\\ D \end{array}\right]$$

where $V_{i}^{d}$ is an *u* × 1 estimator vector for the uncertainty vector *ε*. The covariance matrix of Equation (11) is $\left[\begin{array}{cc} \Sigma_{i} & 0\\ 0 & \Sigma_{d} \end{array}\right]$.

According to the classical Kalman filter theory [8,10,11,12,13,14], the parameter estimate depending on *a priori* distance constraints at epoch *i* can be expressed as

(12) $${\hat{X}}_{i}={\bar{X}}_{i}+\Sigma_{{\bar{X}}_{i}}\left[\begin{array}{cc} A_{i}^{Τ} & B_{i}^{Τ} \end{array}\right]\cdot {\left\{\left[\begin{array}{cc} A_{i}\Sigma_{{\bar{X}}_{i}}A_{i}^{Τ} & A_{i}\Sigma_{{\bar{X}}_{i}}B_{i}^{Τ}\\ B_{i}\Sigma_{{\bar{X}}_{i}}A_{i}^{Τ} & B_{i}\Sigma_{{\bar{X}}_{i}}B_{i}^{Τ} \end{array}\right]+\left[\begin{array}{cc} \Sigma_{i} & 0\\ 0 & \Sigma_{d} \end{array}\right]\right\}}^{-1}\cdot \left[\begin{array}{c} L_{i}-A_{i}{\bar{X}}_{i}\\ D-B_{i}{\bar{X}}_{i} \end{array}\right]$$

and the a posteriori covariance matrix $\Sigma_{{\hat{X}}_{i}}$ is

(13) $$\Sigma_{{\hat{X}}_{i}}=\Sigma_{{\bar{X}}_{i}}-\Sigma_{{\bar{X}}_{i}}\left[\begin{array}{cc} A_{i}^{Τ} & B_{i}^{Τ} \end{array}\right]\cdot {\left\{\left[\begin{array}{cc} A_{i}\Sigma_{{\bar{X}}_{i}}A_{i}^{Τ} & A_{i}\Sigma_{{\bar{X}}_{i}}B_{i}^{Τ}\\ B_{i}\Sigma_{{\bar{X}}_{i}}A_{i}^{Τ} & B_{i}\Sigma_{{\bar{X}}_{i}}B_{i}^{Τ} \end{array}\right]+\left[\begin{array}{cc} \Sigma_{i} & 0\\ 0 & \Sigma_{d} \end{array}\right]\right\}}^{-1}\cdot \left[\begin{array}{c} A_{i}\\ B_{i} \end{array}\right]\Sigma_{{\bar{X}}_{i}}$$

In this study, such a kind of *a priori* distance constraint is developed, which takes into account their actual variance for the GNSS kinematic positioning. In order to achieve this, the accuracy of the measurement of these distances is analyzed and introduced in this approach. This approach can be treated as two groups of observations as well (see, for example, [19]).

In order to illustrate the impact of these distance constraints in GNSS precise positioning for multiple kinematic stations, data of a shipborne gravimetric campaign were used as described in the following section.

## 3. Experiment and Analysis

If the reference stations for a highly dynamic platform are located far away, it is difficult to get sufficiently accurate state information for a kinematic platform using GNSS precise positioning [20]. In order to investigate this context, a benchmark is needed. Therefore, here in our study, GNSS DD positioning results obtained by using the most closely located reference stations are regarded as the “true value” for the comparison with results obtained using reference stations located far away [21]. In this case, the developed method was investigated for long baseline mode for comparison. This method can be applied to airborne and shipborne kinematic platforms. However, it is difficult to get more accurate state information for an airborne platform than such as obtained from GNSS kinematic positioning. Therefore, GNSS data of the shipborne gravimetry campaign on the Baltic Sea were used to illustrate the methodology, since the state information for a kinematic platform from the GNSS ultrashort baselines can be used as a true value to compare it with the state information from GNSS long baseline.

**Figure 2:**
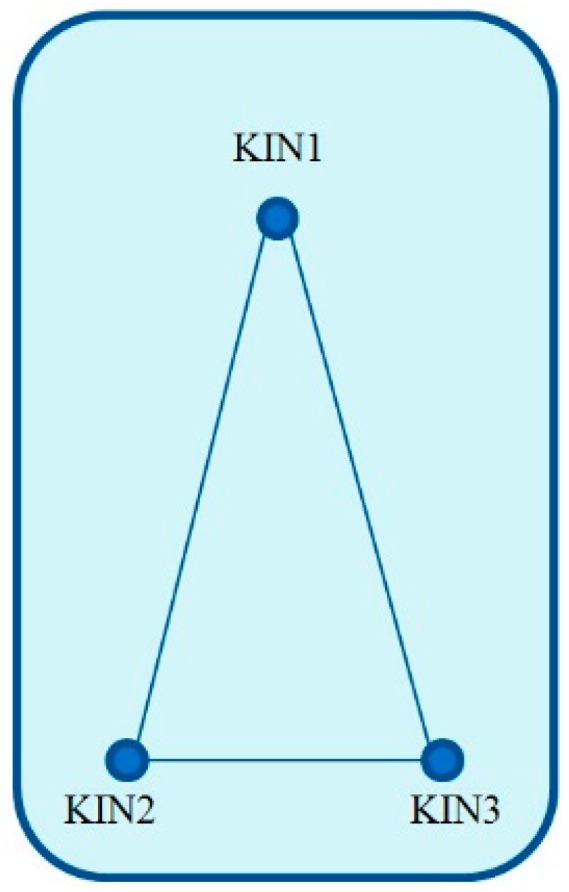
Relative positions of the kinematic GNSS antennas on the ship.

Ten days of GNSS and gravimetric data from 18 to 27 June 2013 were collected by GFZ and BKG in the Baltic Sea near Greifswald, Germany. During this campaign, three GNSS antennas were mounted on the ship. Their relative positions are shown in Figure 2.

**Figure 3:**
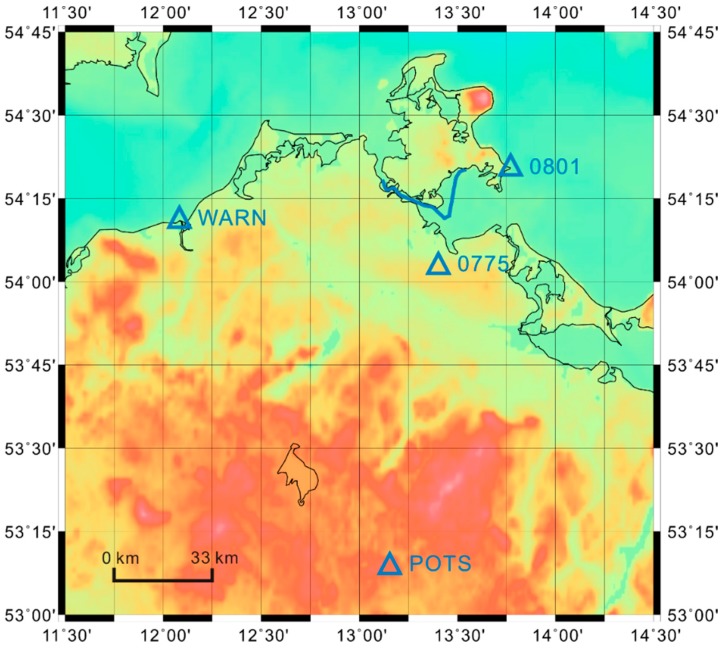
The trajectory of the ship (blue curve) and the positions of all reference stations (blue triangles) of the Baltic Sea shipborne gravimetric campaign on 18 June 2013.

In order to investigate the capability of the new strategy, the GNSS data of the first day (18 June 2013) were selected for testing. The GNSS stations KIN1 and KIN3 were taken as multiple kinematic stations. Their known distance length of 26.342 m was used as distance constraint as described in the following. The stations 0801 and 0775 of the Satellite Positioning Service (SAPOS), which is operated by the Working Committee of the Surveying Agencies of the States of the Federal Republic of Germany (AdV), were taken as nearby-located reference stations (distances < 30 km). Their positions are shown in Figure 3. The IGS stations WARN and POTS were chosen as for-away-located reference stations (distance ~50–200 km). The trajectory of the ship and the positions of the latter reference stations are shown in Figure 3 as well. The hardware types of all GNSS receivers and antennas are given in Table 1.

For the GNSS data processing, the HALO_GNSS software [22] was used for applying the methods described in this study. Here, the dual-frequency carrier phase observations were used to form the ionosphere-free combination. The wet tropospheric zenith path delay was estimated as a random walk process, where the initial uncertainty was assumed to be 10 cm, and its spectral density 10^−12^ m^2^/s. The justifications for the selection of these values were the slow motion of the ship and the small changes in the height profile [9,23]. The two-way Kalman filter [24] was used for the parameter estimation, and the selected data containing GPS and GLONASS observations with a sampling rate of 1 Hz were used for the calculation of the trajectories for the multiple kinematic stations KIN1 and KIN3.

As already explained, in order to demonstrate the capability of our approach, the state information of kinematic platform was first calculated using the nearby reference stations. These results were treated as “true values” for comparison. Then, the state information of the kinematic platform was calculated using the far-away reference stations and was regarded as benchmark. Finally, our new approach with *a priori* constraints was applied to this configuration. The improvement due to the new approach should be obvious from these comparisons. For this purpose, three experimental scenarios were realized with the following computational schemes:

**Scheme 1** (Scenario for the nearby-located reference stations without distance constraints): The trajectories of the multiple kinematic stations KIN1 and KIN3 were calculated, where 0801 and 0775 served as multiple nearby-located reference stations.

**Figure 4:**
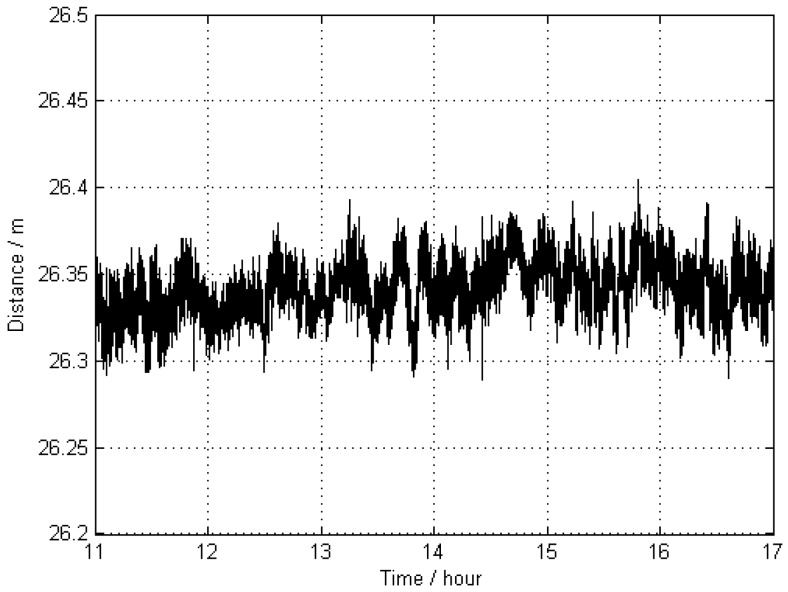
Apparent distance (as a function of time) between two kinematic antennas KIN1 and KIN3 without applying distance constraints; the trajectories of these antennas were estimated by using two reference stations 0801 and 0775 (Scheme 1).

In general, it is difficult to know the true value of the exact position of a moving GNSS antenna. In order to investigate the accuracy of the kinematic results, the distance between the two kinematic stations KIN1 and KIN3 was calculated for each epoch. The apparent changes of this length are shown in Figure 4, and the corresponding statistical results are given in Table 2. Because the results of kinematic GNSS positioning are usually of lower accuracy than those of static GNSS positioning, and this distance as a function of time came from two distinct kinematic antennas, the distance variation of up to 10 cm can be accepted. Therefore, the trajectory as obtained from the kinematic positioning can be treated as a “true value” with a respective standard deviation (STD) of 1.5 cm. Since the results for KIN1 and KIN3 are of almost the same quality, only the results of KIN1 are used for comparisons in the following scenarios.

**Scheme 2** (Scenario with the far-away reference stations without distance constraints): The trajectories of the multiple kinematic stations KIN1 and KIN3 were calculated, where WARN and POTS served as multiple reference stations which are located far away from the kinematic stations.

**Figure 5:**
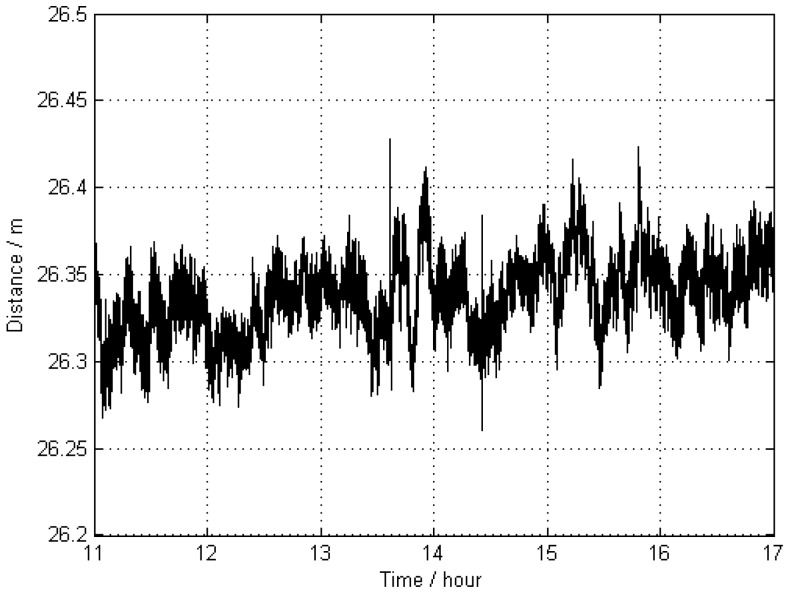
Apparent distance (as a function of time) between the two antennas KIN1 and KIN3 without distance constraints; the trajectories of these antennas were estimated by using two far-away-located reference stations, WARN and POTS (Scheme 2).

The distance between the two kinematic stations KIN1 and KIN3 was calculated for each epoch. The apparent changes of this distance are shown in Figure 5, and the respective statistical results are provided in Table 2. The results demonstrate that the precision of Scheme 2 is lower than that of Scheme 1, since in the case of the far-away reference stations, differential common error residuals increase and may hamper the differential process, or may decrease the accuracy [25,26,27], for instance, due to tropospheric path delay and ionospheric refraction effects.

**Figure 6:**
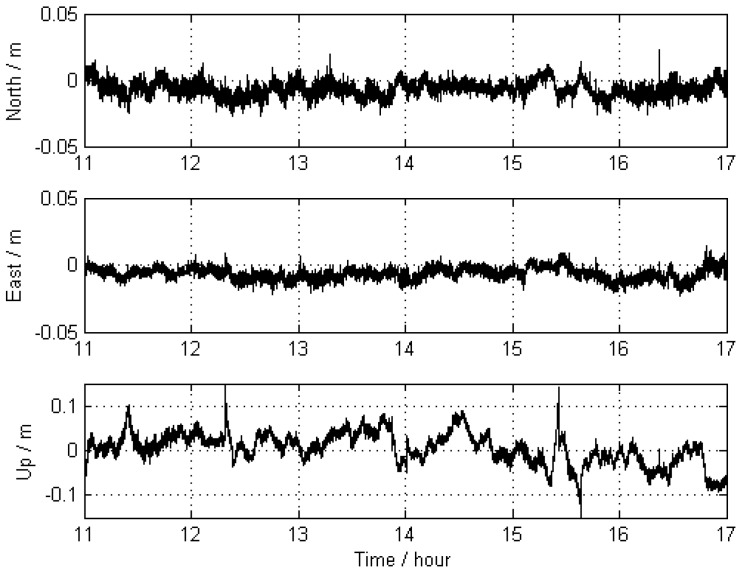
Differences between the trajectories of KIN1 for the scenario with far-away-located reference stations (Scheme 2) and those obtained for Scheme 1.

**Figure 7:**
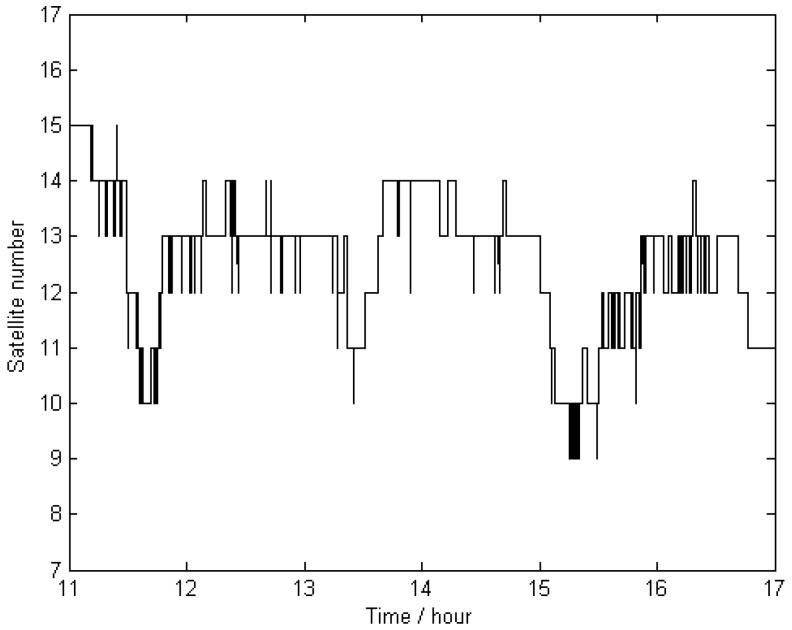
Total number of the visible satellites (GPS + GLONASS) during the measurement time span for this study.

The trajectories of KIN1 for the reference stations located far away were compared with the “true value” of Scheme 1. The comparison results are displayed in Figure 6, and their statistics are given in Table 3, showing that the achieved accuracy (in terms of RMS) is 5.8 mm, 4.5 mm, and 36.8 mm for the north, east, and up directions, respectively. The large deflections that appear in Figure 6 between hours 15 and 16 are obviously caused by a lower number of visible satellites at that time. The total number of visible satellites (GPS plus GLONASS) is shown in Figure 7.

**Scheme 3:** Based on Scheme 2, the *a priori* distance constraint was applied when calculating the trajectories of KIN1 and KIN3.

**Figure 8:**
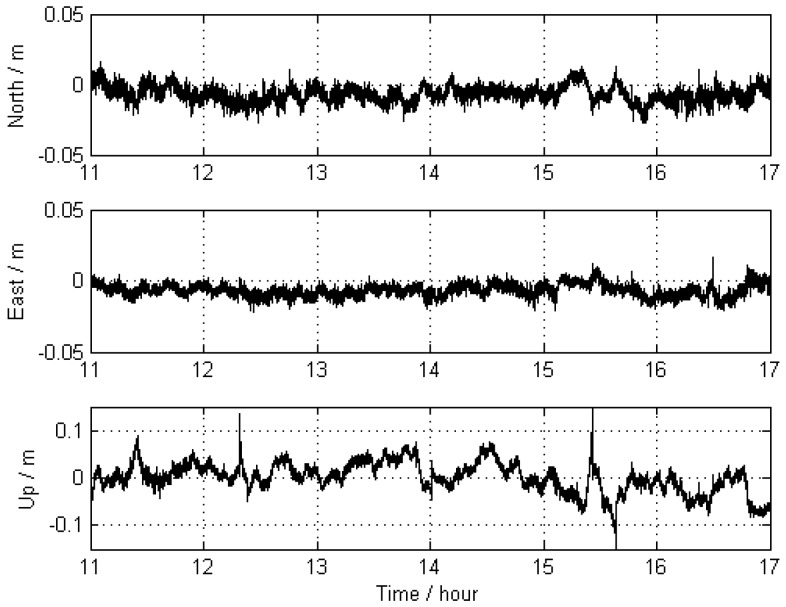
Differences between the KIN1 positioning results obtained from Scheme 3 (far-away-located reference stations and distance constraints) and those of Scheme 1.

The trajectory of KIN1 was compared with the “true value” of Scheme 1 as well. The differences of the positions are shown in Figure 8, and corresponding statistical results are given in Table 3, showing that the achieved accuracy is 5.8 mm, 4.2 mm, and 33.1 mm for the north, east, and up directions, respectively. In comparison with Scheme 2, the new approach obviously improves the accuracy of the GNSS kinematic positioning by 0.3 mm and 3.7 mm for the east and up directions.

From the obtained computational results, the following conclusions can be drawn:

When applying the method of GNSS kinematic positioning (based on DD) for multiple kinematic stations and multiple reference stations, the precision of the state estimates of the kinematic stations when using far-away-located reference stations (Scheme 2) is lower than that obtained by the usage of nearby-located reference stations (Scheme 1), since, in the first case, differential common residual errors increase and may hamper the differential process, or may decrease the accuracy.

Because of the relationships or known information between state parameters used in GNSS precise kinematic positioning, the accuracies of the estimated kinematic state parameters can be improved by applying *a priori* distance constraints, especially in the up direction. It is worth pointing out that, in this comparison, uncertainties occurs for both nearby and far-away reference stations, and it is difficult to obtain a "true value" for the time variable position of a kinematic platform. Nevertheless, this comparison method illustrates the performance of the approach applied in this study.

## 4. Conclusions

In order to improve the accuracy of GNSS kinematic positioning for a kinematic platform, a new approach was proposed based on multiple GNSS receiving equipment mounted on this platform. In our study, we make use of the distances among multiple mechanically fixed GNSS antennas, which are usually known and invariant. Therefore, this information can be used as *a priori* distance constraints to improve the RMS accuracy of the state estimates. We propose a special method for such *a priori* distance constraints by considering the accuracy of the measured distance between the multiple GNSS antennas when applying the constraints. Finally, the GNSS data of a shipborne gravimetric campaign in the Baltic Sea was used to test this method, and the results show that the accuracies of the estimated kinematic state parameters, using the *a priori* distance constraints, can be obviously improved, especially in the up direction (an improvement of 3.7 mm).

## Figures and Tables

**Table 1:** Hardware equipment of the chosen stations from the Baltic Sea gravimetric campaign.

Station Name	Receiver Type	Antenna Type	With Radome
KIN1	JAVAD TRE_G3TH DELTA	LEIAS10	NONE
KIN3	JAVAD TRE_G3TH DELTA	ACCG5ANT_42AT1	NONE
0801	TPS NET-G3A	TPSCR.G3	TPSH
0775	TPS NET-G3A	TPSCR.G3	TPSH
WARN	JPS LEGACY	LEIAR25.R3	LEIT
POTS	JAVAD TRE_G3TH DELTA	JAV_RINGANT_G3T	NONE

**Table 2:** The statistical results for the distance between KIN1 and KIN3 (Unit: m).

Scheme	Reference Stations	Min	Max	Mean	STD
1	Nearby	26.282	26.406	26.342	0.015
2	Far away	26.261	26.428	26.338	0.022

**Table 3:** Statistics for the differences between the positioning results for KIN1 obtained from Scheme 2 resp. 3 , and the corresponding results from Scheme 1 (Unit: mm).

Scheme	Direction	Min	Max	Mean	RMS
**2 vs. 1**	North	–27.0	23.0	–6.4	5.8
	East	–23.5	14.8	–6.4	4.5
	Up	–165.7	165.9	3.5	36.8
**3 vs. 1**	North	–27.7	15.8	–6.4	5.8
	East	–21.6	16.4	–6.6	4.2
	Up	–161.6	145.4	0.0	33.1
